# Inferring Correlation Networks from Genomic Survey Data

**DOI:** 10.1371/journal.pcbi.1002687

**Published:** 2012-09-20

**Authors:** Jonathan Friedman, Eric J. Alm

**Affiliations:** 1 Computational & Systems Biology Initiative, Massachusetts Institute of Technology, Cambridge, Massachusetts, United States of America; 2 Departments of Biological Engineering & Civil and Environmental Engineering, Massachusetts Institute of Technology, Cambridge, Massachusetts, United States of America; 3 The Broad Institute, Cambridge, Massachusetts, United States of America; University of Zurich and Swiss Institute of Bioinformatics, Switzerland

## Abstract

High-throughput sequencing based techniques, such as 16S rRNA gene profiling, have the potential to elucidate the complex inner workings of natural microbial communities - be they from the world's oceans or the human gut. A key step in exploring such data is the identification of dependencies between members of these communities, which is commonly achieved by correlation analysis. However, it has been known since the days of Karl Pearson that the analysis of the type of data generated by such techniques (referred to as compositional data) can produce unreliable results since the observed data take the form of relative fractions of genes or species, rather than their absolute abundances. Using simulated and real data from the Human Microbiome Project, we show that such compositional effects can be widespread and severe: in some real data sets many of the correlations among taxa can be artifactual, and true correlations may even appear with opposite sign. Additionally, we show that community diversity is the key factor that modulates the acuteness of such compositional effects, and develop a new approach, called SparCC (available at https://bitbucket.org/yonatanf/sparcc), which is capable of estimating correlation values from compositional data. To illustrate a potential application of SparCC, we infer a rich ecological network connecting hundreds of interacting species across 18 sites on the human body. Using the SparCC network as a reference, we estimated that the standard approach yields 3 spurious species-species interactions for each true interaction and misses 60% of the true interactions in the human microbiome data, and, as predicted, most of the erroneous links are found in the samples with the lowest diversity.

## Author Summary

Genomic survey data, such as those obtained from 16S rRNA gene sequencing, are subject to underappreciated mathematical difficulties that can undermine standard data analysis techniques. We show that these effects can lead to erroneous correlations among taxa within the human microbiome despite the statistical significance of the associations. To overcome these difficulties, we developed SparCC; a novel procedure, tailored to the properties of genomic survey data, that allow inference of correlations between genes or species. We use SparCC to elucidate networks of interaction among microbial species living in or on the human body.

## Introduction

The study of natural communities using high throughput genomic surveys, such as 16S rRNA gene profiling, has become routine [1], yet the development of appropriate, well validated analysis methods is still ongoing. The first challenge is obtaining reliable and informative counts from 16S rRNA gene sequences by filtering spurious reads and grouping the remaining reads in a meaningful way [2], [3], [4]. Once such counts have been obtained, analysis techniques which are appropriate for discrete survey data need to be applied [5], [6] [7].

A common goal of genomic surveys is to identify correlations between taxa within ecological communities. Correlation analysis provides a well trodden path to achieving this goal, but we show that it is not valid when applied to genomic survey data (GSD), and may produce misleading results. The challanges associated with GSD stem from the fact that they are a relative, rather than absolute, measure of abundances of community components. The counts comprising these data (e.g., 16S rRNA gene reads) are set by the amount of genetic material extracted from the community or the sequencing depth, and analysis typically begins by normalizing the observed counts by the total number of counts. The resulting fractions fall into a class of data termed closed or compositional, and poses its particular geometrical and statistical properties [8], [7]. Specifically, standard methods for computing correlations from GSD are theoretically invalid. Correlation estimates are biased by the fact that, since they must sum to 1, fractions are not independent and tend to have a negative correlation regardless of the true correlation between the underlying absolute abundances (termed the basis abundances) [9]. Thus, correlations estimates often reflect the compositional nature of the data, and are not indicative of the underlying biological processes [10]. In fact, in 1897 Karl Pearson warned against “attempts to interpret correlations between ratios whose numerators and denominators contain common parts” [11], and since that time it has been shown that many other standard analysis techniques are invalid when applied to such compositional data, and that their interpretation is unreliable and often misleading [10], [12], [13]. Nonetheless, these methods remain the primary tools used in studies of microbial ecology.

Although approaches to compositional data analysis have been developed (e.g. [13], [14]), the basic task of inferring dependencies between components remains an outstanding challenge. A widely used method is Aitchison's test for complete subcompositional independence [15], which tests whether any dependencies are present, but does not indicate which components are correlated, nor the magnitude of the correlation. Filzmoser and Hron [16] recently developed a method for inferring correlations in compositional data after an appropriate mathematical transformation, but their method does not provide a mapping relating the correlations of the transformed variables to those of the underlying genes or species.

In this paper, we first use simulations and real-world data from the Human Microbiome Project (HMP) to demonstrate that GSD can be severely biased by “compositional” effects, and then identify the factors the modulate their severity. Finally, we present a novel method, called SparCC, and show that it can infer correlations with high accuracy even in the most challenging data sets.

## Results

### Standard correlation inference techniques perform poorly on GSD

To what extent do compositional artifacts affect real-world GSD? We applied standard statistical methods to 16S rRNA gene survey data from the Human Microbiome Project (HMP) [17], which measure the compositions of microbial communities found in different body sites of 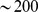 individuals. The composition of each community is described in terms of operational taxonomic units (OTUs). Because only relative abundances for each OTU are available, these data qualify as compositional and are thus subject to potential biases as described above.

**Figure 1:**
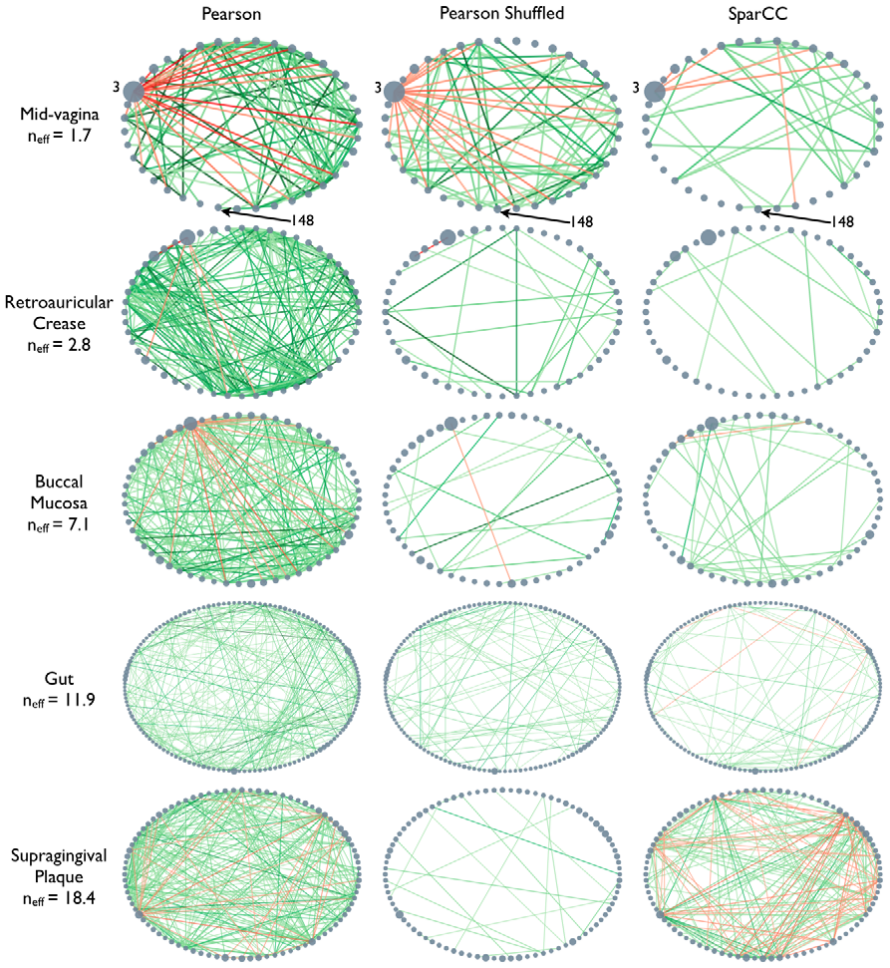
Similar correlation networks are observed for real world vs. randomly shuffled bacterial abundance data. Correlation networks based on 16S rRNA gene survey data collected as part of the Human Microbiome Project (HMP), inferred using Pearson correlations (left column), and SparCC (right column). Additionally, Pearson correlation networks were inferred from shuffled HMP data (middle column), where all OTUs are independent. The Pearson networks inferred from shuffled data show patterns similar to the ones seen in the Pearson networks of the real data, especially for low diversity body sites. This indicates that the observed Pearson network structure may be due to biases inherent in compositional data rather than a real biological signal. In contrast, no significant correlation were inferred from the shuffled data using SparCC (data not shown). Nodes represent OTUs, with size reflecting the OTU's average fraction in the community. Edges between nodes represent correlations between the nodes they connect, with edge width and shade indicating the correlation magnitude, and green and red colors indicating positive and negative correlations, respectively. For clarity, only edges corresponding to correlations whose magnitude is greater than 0.3 are drawn. See Fig. S1 for all 18 HMP body sites.

Networks inferred from Standard Pearson correlation display distinct patterns within different body sites, suggestive of biological structure (Fig. 1, left column. See Fig. S1 for all 18 HMP body sites). Specifically, a prominent feature of the mid-vagina, retroauricular crease, and buccal mucosa networks is the presence of an OTU that is negatively correlated with multiple other OTUs. Despite the temptation to attribute biological significance to these observations, correlation networks inferred from randomly shuffled data with similar taxon abundances, but lacking any correlations between OTUs (see Materials and Methods), reproduce this feature (Fig. 1, middle column) indicating that it may arise from the closure (normalization) process.

The mechanism behind these spurious correlations is straightforward. The pattern observed in the mid-vagina network results from the dominance of OTU 3, a *Lactobacillus*. This OTU has a median abundance of 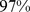, so fluctuations in its relative abundance have a strong effect on the abundance of the rest of the community simply due to the requirement that the relative abundances of all OTUs sum to 100%: when the abundance of *Lactobacillus* varies, all other OTUs' relative abundances vary in unison in the opposite direction creating artificial negative correlations with *Lactobacillus*, and artificial positive correlations with each other.

### Diversity and correlation density control the severity of compositional effects

Compositional effects are severe in some datasets, but mild in others. We found that diversity of the samples in the dataset (often referred to as alpha diversity), is a good predictor of the strength of compositional effects, which diminish with increased diversity. Intuitively, the fewer OTUs comprise the community, the worse the compositional effects are, with the extreme case of a community composed of only two OTUs, which will always appear to be perfectly negatively correlated. Moreover, compositional effects can be significant even in communities comprised of multiple OTUs, if only a few OTUs dominate the community. This notion of diversity can be quantified using the Shannon effective number of OTUs,(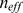) [18], which quantifies both the number of OTUs and the dominance in a community. 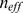 ranges from 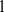, when the community is completely dominated by a single OTU, to the number of OTUs in the community (richness), when all OTUs are equally abundant.

**Figure 2:**
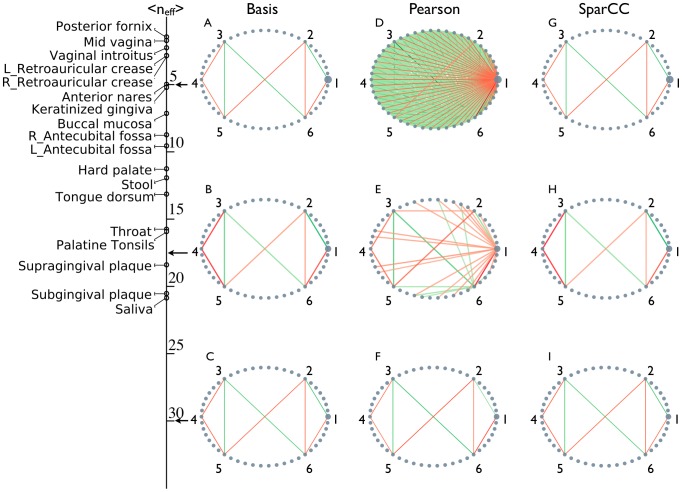
Pearson correlations inference quality deteriorates with decreasing diversity. Basis data was simulated with a known correlation structure. OTU counts were generated by randomly drawing from the basis, and were subsequently subject to both correlation inference procedures. (A–C) True basis correlation network. (D–F) Networks inferred using standard procedure. (G–I) Networks inferred using SparCC. The average community diversities, as given by the Shannon entropy effective number of components 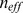, used in the simulations and observed in the HMP data are indicated on left indicates. As in Fig. 1, nodes represent OTUs, with size reflecting the OTU's average fraction in the community. Nodes represent OTUs, with size reflecting the OTU's average fraction in the community. Edges between nodes represent correlations between the nodes they connect, with edge width and shade indicating the correlation magnitude, and green and red colors indicating positive and negative correlations, respectively. For clarity, only edges corresponding to correlations whose magnitude is greater than 0.3 are drawn.

Simulated networks of varying 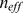 (see Material and Methods) with known correlations illustrate the effect of diversity on compositional artifacts. True correlations (Fig. 2A–C) are only recovered when the community is diverse (Fig. 2F). In networks of similar diversity to the HMP samples, inferred connections are often dominated by negative correlations to the dominant OTU, which leads to positive correlations among the remaining OTUs (Fig. 2D,E). This effect is so strong that it eliminates the negative correlation between OTU 4 and OTUs 3 and 5, and positive correlation between OTUs 1 and 2 (Fig. 2E). Worse yet, as diversity decreases further, the negative correlation between OTU 4 and OTUs 3 and 5 is turned into an apparently positive one (Fig. 2D). It is important to note that these compositional effects are not limited to Pearson correlation, and are also present in non-parametric correlations, such as Spearman correlations (Fig. S2).

If the underlying network has true positive correlations, then compositional effects can be even more pronounced than expected based on the community diversity. This happens because strong correlations between components lowers the effective diversity of the sample (i.e., two OTUs that are perfectly correlated behave as a single OTU). This effect can confound naive efforts to correct for compositional effects by comparing observed correlations against shuffled networks. When the data are shuffled, as in the middle column of Fig. 1, few spurious connections may arise relative to the structure observed for the unshuffled data (as observed for the buccal mucosa samples), creating false confidence in the observed network. Thus, randomization is not sufficent to establish significance of observed correlations, nor is it possible to identify correlations by comparing against (or “subtracting out”) a randomized network.

### SparCC: a novel procedure for inferring correlations from GSD

Here, we describe a new technique for inferring correlations from compositional data called SparCC (Sparse Correlations for Compositional data). SparCC estimates the linear Pearson correlations between the log-transformed components. Since these correlations cannot be computed exactly (as described below), SparCC utilizes an approximation which is based on the assumptions that: (i) the number of different components (e.g., OTUs or genes) is large, and (ii) the true correlation network is ‘sparse’ (i.e., most components are not strongly correlated with each other). Later, we show that SparCC is surprisingly robust to violations of the sparsity assumption. SparCC does not rely on any particular distribution of the basis variables, i.e. the true abundances in the community can follow any distribution, and the choice of the log-normal distribution in subsequent examples is motivated solely by ease of implementation and empirical fit. For clarity, we present the method in the context of 16S rRNA gene data, where the components are OTUs and the basis variables are their true abundances in a community, but SparCC can be applied to any compositional data for which its approximation is valid.

Like most compositional data analysis techniques, SparCC is based on the log-ratio transformation:
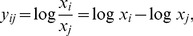
(1)where 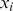 is the fraction of OTU 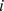. This transformation carries several advantages: First, the new variables 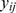 contain information regarding the true abundances of OTUs, as the ratio of fractions is equal to the ratio of the true abundances. Second, unlike the fractions themselves, the ratio of the fractions of two OTUs is independent of which other OTUs are included in the analysis, a property termed subcompositional coherence. Third, this transformation is mathematically convenient, as the new variables 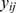 are no longer limited to the simplex, but are free to assume any real value. Taking the logarithm removed the positivity constraint, and induces (anti) symmetry in the treatment of the variables.

To describe the dependencies in a compositional dataset, Aitchison suggested using the quantity
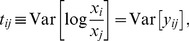
(2)where the variance is taken across all samples [12]. When OTUs are perfectly correlated, their ratio is constant, therefore 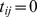, whereas the ratio of uncorrelated OTUs varies and the corresponding 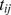 is large. Though 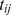 contains information regarding the dependence between the OTUs, it is hard to interpret as it lacks a scale. That is, it is unclear what constitutes a large or small value of 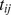 (does a value of 0.1 indicate strong dependence, weak dependence, or no dependence?). This can be further appreciated by relating 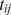 to our quantity of interest, the correlation between the true abundances of the OTUs. The relation is given by
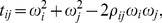
(3)where 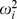 and 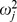 are the variances of the log-transformed basis abundances of OTUs 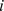 and 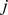, and 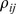 is the correlation between them. It is now evident that 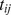 can only be interpreted in relation to the basis abundance's variances: 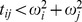 indicates a positive correlation, and 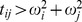 indicates a negative correlation. Ideally, we would like to solve the set of eqs. 3 for all OTU pairs and simultaneously infer both the basis variances and correlations. However, because there are more unknown variables than equations, this is not generally possible. Nonetheless, it is possible to obtain a good approximation of the variances if, on average, OTUs are uncorrelated. Once we obtain estimates of the basis variances, these can be plugged into eqs. 3 to infer the correlations between each OTU pair, which, unlike the average correlations, needn't be small.

More accurate estimation can be achieved by iterating the above procedure. At each iteration the strongest correlated OTU pair identified in the previous iteration is excluded from the basis variance estimation. This reinforces sparsity among the remaining pairs and yields better variance and correlation estimates.

OTU fractions need to be estimated from the observed counts to apply SparCC. Normalizing each OTU by the total counts in the sample (the maximum-likelihood estimate) is unreliable for rare OTU because it overestimates the number of zero fractions [19]. This can give rise to artifacts that are driven by variations in the sequencing depth. These artifacts have motivated some authors to downsample their data such that all samples have the same total counts, however downsampling does nothing to alleviate compositional effects, and requires discarding a substantial portion of the available data. Therefore, we employed a Bayesian approach to estimate component fractions (see Materials and Methods), which allows the assessment of the robustness of downstream analysis and the assignment of confidence values.

### SparCC is highly accurate on simulated data

**Figure 3:**
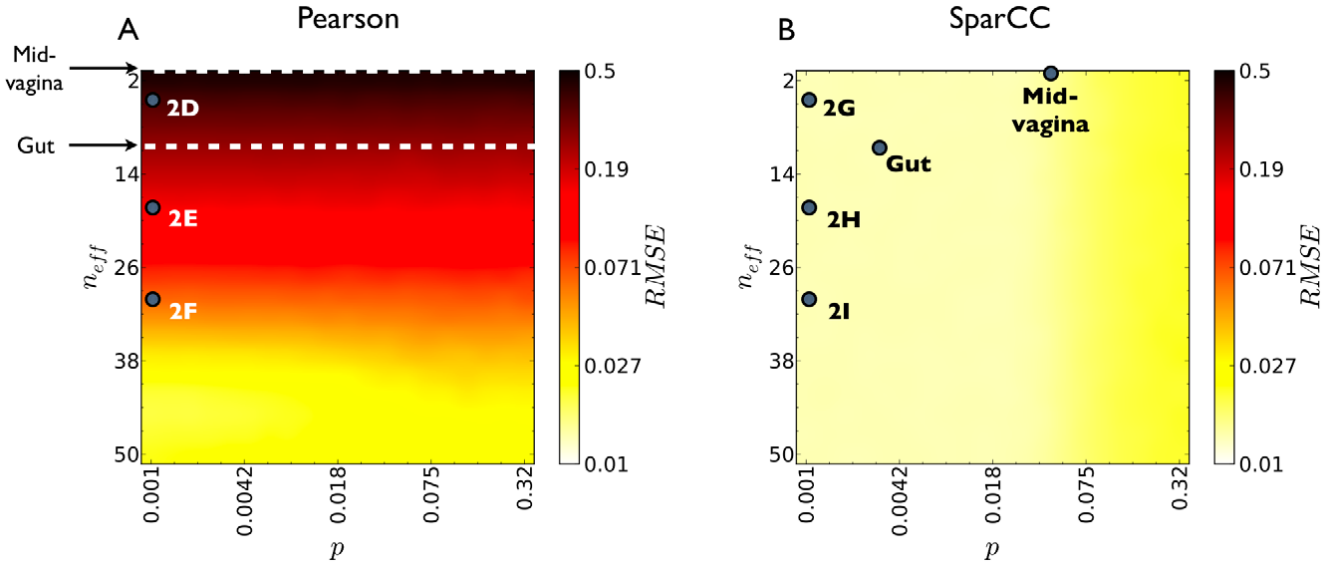
SparCC outperforms standard inference. Root-mean-square error (RMSE) of both Pearson (A) and SparCC (B) inferred correlations, as a function of the density of the underlying correlation network, as given by the probability that any pair of components be strongly correlated 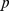, and community diversity, as given by the Shannon entropy effective number of components 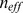. SparCC errors are smaller than Pearson errors for all parameter values. For the maximal diversity plotted, 50 effective OTU, the inference error obtained using Pearson correlations is greatly decreased. Therefore, it is likely that Pearson correlations perform well on gene expression data, where the effective number of genes is typically in the hundreds or thousands. For each combination of density and diversity, multiple basis correlation networks were randomly generated, and corresponding data was sampled and used for correlation estimation. Dots labeled mid-vagina and gut indicate the average diversity observed in the mid-vagina and gut communities, and the density of their estimated correlation networks. Dots labeled 2D–I indicate the diversity and density used to generate the communities analyzed in Fig. 2.

We used the previously described simulated datasets to demonstrate the accuracy of SparCC at inferring correlations, even in highly problematic compositional data dominated by a single OTU (Fig. 2G–I). A more systematic evaluation of SparCC was performed by creating multiple simulated datasets of varying diversity and density. We measure density as the average Pearson correlation between OTUs, such that denser datasets have more strongly correlated OTUs, challenging the sparsity assumption used by SparCC. For each combination of density and diversity, multiple true correlation networks were assigned, and corresponding data was sampled. Networks inferred by SparCC or standard correlations were evaluated using the root-mean-square error (RMSE) (Fig. 3). Standard techniques only gave reasonable estimates for very diverse, sparse networks (Pearson RMSE 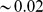), whereas for networks with diversity comparable to those observed in the HMP set, the Pearson RMSE was unacceptably high, reaching 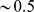 for communities with diversity similar to the mid-vagina. Spearman correlations performed only marginally better (Fig. S3A). By contrast, the performance of SparCC was independent of diversity, and gave improved results for all parameter values, even for dense networks in which the sparsity assumption is violated. In fact, the worst accuracy achieved by SparCC (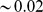, for unrealistically dense networks), was comparable to the best accuracy achieved using standard correlations on highly diverse samples. Moreover, though stronger correlation can be estimated more reliably, using standard methods, attention needs to be restricted to exceptionally strong correlations before the accuracy improves significantly, and the resulting accuracy is at best comparable to SparCC's accuracy (Fig. S5).

### SparCC identifies phylogenetically structured correlations in HMP data

**Figure 4:**
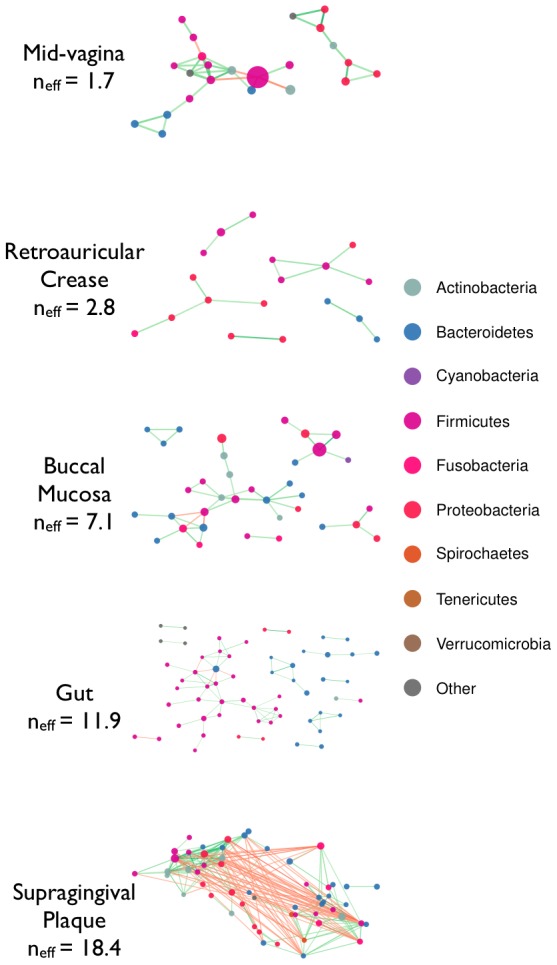
HMP correlation networks inferred using SparCC. Networks inferred using SparCC from the same data as in Fig. 1 (see Fig. S2 for SparCC networks of all HMP body sites). No correlations with magnitude greater than the 0.3 cutoff were inferred from the shuffled data (not shown). Nodes represent OTUs, with size reflecting the OTU's average fraction in the community, and color corresponding to the phylum to which the OTU belongs. Edges between nodes represent correlations between the nodes they connect, with edge width and shade indicating the correlation magnitude, and green and red colors indicating positive and negative correlations, respectively. For clarity, only edges corresponding to correlations whose magnitude is greater than 0.3 are drawn, and unconnected nodes are omitted. See Fig. S6 for all 18 HMP body sites.

We used SparCC to infer the taxon-taxon interaction networks from the HMP data sets (Fig. 1, right column, Fig. 4), and from their corresponding shuffled datasets (in which all OTUs are uncorrelated). In contrast to the naive approach shown in Fig. 1, SparCC found no significant correlations in the shuffled dataset (Dataset S1). For the real data, however, numerous correlations are found, which differed significantly from the standard Pearson correlations. SparCC inference indicated that on average 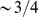 of the correlated OTU pairs identified using Pearson were false, and that 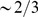 of the correlated OTU pairs were missed using Pearson (see Table S1 for breakdown by body site.). Of particular note, we observe a positive correlation between OTU 3 and OTU 148, both belonging to the *Lactobacillus* genus, which was absent from the Pearson network, likely because of the bias of the highly abundant OTU 3 toward making negative correlations. Intriguingly, using SparCC we observe a higher likelihood of positive correlations between phylogenetically related taxa (Table S2), a finding that on its surface seems to support a role for neutral community dynamics as related organisms are likely to inhabit similar niches, but do not seem to dominate by competitive exclusion (although more complicated scenarios are certainly possible). We anticipate that techniques such as SparCC will play a major role in analyzing these data to address this and other basic ecological questions.

## Discussion

In this study we have focused on an outstanding challenge of compositional data analysis – inference of correlations. We have demonstrated that compositional effects are pronounced in 16S rRNA gene surveys of the human microbiome, and, motivated by the properties of this data, have developed a novel procedure for estimating correlations.

We found that diversity of species and density of interactions are the two key factors that influence the severity of compositional effects on correlation estimates, with low diversity, high density data being the most challenging to infer correlation from using standard methods. SparCC does not rely on high diversity, rather it only requires sparsity of correlations, but in practice is robust even when the sparsity assumption is strongly violated (%30 of all component pairs are strongly correlated). Therefore, we recommend that SparCC be used on any GSD that has low diversity: as a rule of thumb we recommend an effective number of components of at least 50 for standard techniques (with the potential caveat that if strong positive correlations are present among many OTUs, the effective diversity may be much lower than estimated). We emphasize that simply having many components is not sufficient to avoid compositional effects. For example, 16S rRNA gene surveys from the HMP include hundreds to thousands of distinct OTUs, yet have have a relatively low effective number of species, with a small number of species dominating most samples.

An important subclass of GSD are genome-wide surveys conducted using techniques such as DNA microarrays, RNA-seq and ChIP-seq. These genome-wide data are also subject to compositional effects, however, as these data tend to have high diversity, they are likely to be much less severe or negligible. For example, the average effective number of genes in microarray experiments available through the 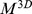 database [20] was 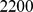 for *S. cerevisiae* and 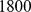 for *E. coli*. This may explain why to date comparatively less attention has been paid to compositional effects in the biological sciences than in other disciplines.

The preponderance of zero values are another area of concern with GSD. These zeros can represent either components that are truly absent from the community, or rare components that, by chance, were not present in the sample drawn from the community. Without additional knowledge, these options are indistinguishable, and, depending on goal of the analysis, the researcher must decide how to interpret them, and choose analysis methods accordingly. We emphasize that the treatment of zero values is a challenge that is in no way unique to compositional data, but is merely highlighted by the log-ratio transformations employed to analyze these data [21]. In this study, we eliminate zero fractions by adding small pseudocounts, as detailed in the Materials and Methods. Complementary approaches, where zeros are treated differently than non-zero values, are substantially more challenging, and are the subject of ongoing research [22].

Though the method presented in this paper allows detection of correlation within communities, many challenges still remain. First, SparCC relies on having reliable component counts, which as noted in the introduction, is not trivial. Second, the correlations estimated by SparCC measure the linear relationship between log transformed abundances. Compositional methods for inferring more general dependencies between components, equivalent to rank correlations and mutual information for non-compositional data, have not yet been developed. Third, relating the patterns detected within a community to external factors (e.g. relating the composition of a human gut microbial community to human health status), and detecting temporal patterns within and between communities requires non-standard, compositional approaches. While some such methods exist [13], [12], [23] they are rarely employed in the context of GSD, and are not tailored for its particular properties. Finally, GSD is often associated with phylogenetic information (relatedness of species or genes), which ideally would be included in the analysis (e.g. the weighted UniFrac distance, which attempts to capture differences in both abundance and phylogenetic composition of communities.). We believe that developing systematic, statistically-sound methods for such analyses of compositional GSD is a necessary step on the road to understanding the structure of biological communities, the processes by which they evolve, and the forces that shape them, and thus represents an important direction for future research.

## Materials and Methods

### HMP 16S rRNA gene data

HMP OTU counts and their taxonomic classification were obtained from the HMPOC dataset, build 1.0, available at http://hmpdacc.org/ [24]. The dataset corresponding to high-quality reads from the v3–5 region was used. Only samples from the May 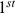 production study were included in the analysis. Additionally, if multiple samples were obtained from the same body site of an individual, only the first sample collected was included in the analysis. For each body site, the data was further filtered by removing samples for which less than 500 reads were collected and OTUs that were, on average, represented by less than 2 reads per sample.

### Shuffled HMP datasets

Shuffled datasets are created by assigning each OTU in each sample a number of counts that is randomly sampled from the OTU's observed counts across all samples, with replacement. This procedure ensures that the resulting marginal distributions of counts of each OTU alone are the same as in the real data, and that there are no correlations between the OTUs in the simulated data.

### Simulated basis datasets for basis correlations estimation

Simulated communities were generated by sampling the joint abundances of 50 OTUs from a log-normal distribution with a given mean and covariance matrix. The mean abundances were equal for all OTUs except OTU 1, whose abundance was set such that the community will have a given effective number of OTUs (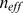), on average. The variance was set to 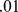 for all OTUs, and random covariance matrices were generated by assigning each OTU pair a probability 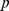 of being perfectly correlated, with positive or negative correlations being equally probable. The resulting random symmetric matrix was then converted to the nearest positive-definite matrix to ensure it is a valid covariance matrix. 500 individuals were randomly sampled from each of these communities to give counts data similar to the one contained in GSD.

For each combination of the parameters 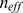 and 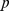, 50 such random communities were simulated, and the correlation inference accuracy was quantified using the root-mean-squared error averaged over all OTU pairs, given by:
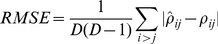
(4)The final inference error is given by averaging the inference error of all 50 runs.

### Effective number of species

The entropy effective number of species of a community, is defined as
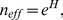
(5)where 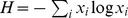 is the entropy of the community [18]. Sample entropies were computed according to the method descried by Chao and Shen [25], as implemented in the R ‘entropy’ package [26]. For each body-site, the effective number of species reported in the main text is the average of the effective number of species of all samples corresponding to that body-site.

### Estimation of component fractions

We adopt a bayesian framework for estimating the true fractions from the observed counts. Assuming unbiased sampling in the sequencing procedure, and a uniform prior, the posterior joint fractions distribution is the Dirichlet distribution [27]:
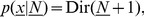
(6)where 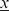 and 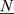 are vectors of the components' true fractions and observed counts, respectively. Unlike Maximum-Likelihood estimation, the bayesian approach results in the full joint distribution of fractions, rather than their point estimates.

Point estimates of fraction values, if desired, can be given by the the mean of the posterior distribution:
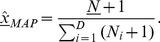
(7)which is equivalent to adding a pseudocount of 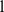 to all count values, and normalize by the total number of counts in each sample. However, we prefer setting the estimator of true fractions to be a random sample from this posterior distribution. This randomness avoids the detection of spurious correlations between rare components, which arrises since the fractions resulting from adding a fixed value pseudocount mirror the sampling depth. Additionally, repeating downstream analysis using many such randomly drawn estimators allows the quantification of the effects of sampling noise on the analysis (one can attempt to model the noise analytically, but this often challenging in practice).

It is important to note that in SparCC, like in any method employing log transformations, some pre-processing is required to eliminate zero values. As described above, SparCC employes a variation of the well-known pseudocounts method which assigns a small fraction to OTUs that were not detected in a sample. This approach implicitly assumes that all components are in fact present in the sample, and that all zero value result from finite detection resolution [19]. For very rare OTUs who are only present at a few samples, this may not be a reasonable assumption. Even if this assumption holds, typically there is not enough information to reliably estimate correlations involving such components, and such components should not be included in the correlation analysis.

### Basic SparCC

As noted in the main text, the quantity
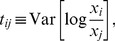
(8)contains information about the dependence between components 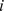 and 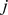, and can be related to the basis correlations. The relation is obtained
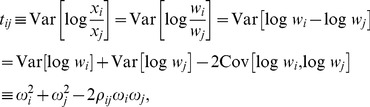
(9)where 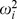 and 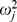 are the variances of the log-transformed basis variables 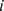 and 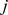, and 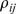 is the correlation between them [10]. Our aim is to exploit relation 9 to infer the unobserved covariance matrix of the log transformed basis variables 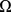, from Aitchison's variation matrix 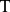, whose elements are 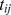. Unfortunately, this is impossible for the most general case, since the basis variances are unknown a priori, and the system of equations for all pairs of components is underdetermined, as it involves 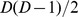 equations and 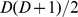 variables (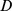 variances and 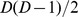 correlations). In fact, even the 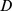 variance variables alone, with all correlations set to zero, allow solving eq. 9 for up to three components. Therefore, at least four components are required to detect deviations from complete independence between all components (this is related to the fact that Aitchison's test for complete subcompositional independence is only effective when at least four components are analyzed [28]).

Since an exact solution cannot be found, we SparCC utilizes an approximation, which is valid when there are many components which are only sparsely correlated. Eq. 9 can be rearranged to give the following expression for the correlation:
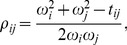
(10)which, given the basis variances can be solved to give the basis correlations. Therefore, we employ the following approximation procedure to estimate the basis variances: First, define the variation of component 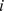 as
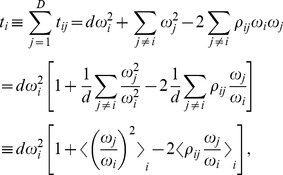
(11)where 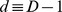, and 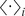 represents averaging over all pairs involving component 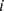. Next, assume that the correlation terms in eq. 11 are small, i.e.
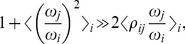
(12)and neglect them, yielding the approximate set of equations:
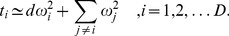
(13)Finally, solve eq. 13 to obtain the approximated basis variances to be plugged into eq. 10, yielding values of the basis correlations.

To elucidate the nature of this approximation, consider the case where all the basis variables have the same variance 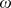. The assumption made in eq. 12 simplifies to:
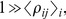
(14)i.e., we assume that the average correlations are small, rather than requiring that any particular correlation be small.

Using the above approximation, the basic inference procedure is the following:

1. Estimate the component fractions in all the samples as outlined above, to obtain the fractions matrix 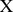.
2. Compute the variation matrix 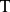.
3. Compute the component variations 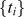.
4. Solve eqs. 13 to get an approximate value for all basis variances 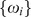.
5. Plug the estimated log-basis variances into eqs. 9 to obtain the basis correlations 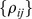.

### Iterative SparCC

**Figure 5:**
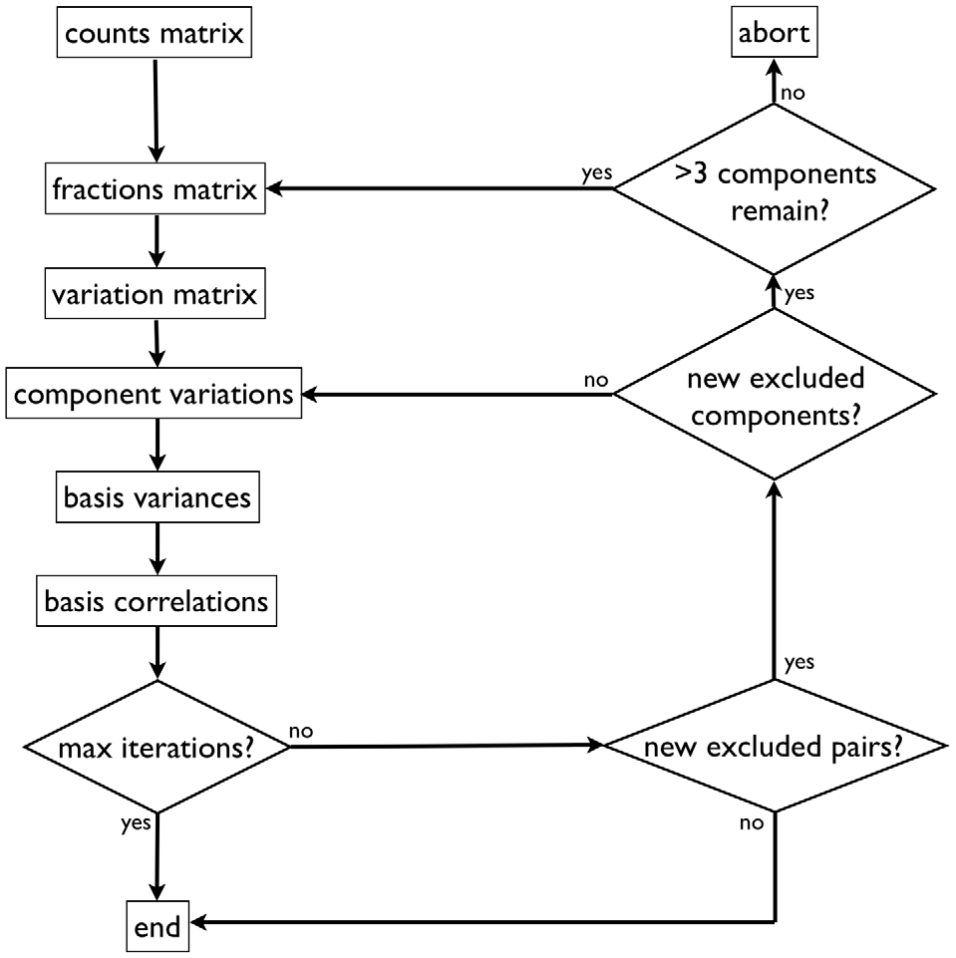
Flow chart of iterative basis correlation inference procedure.

The basic inference procedure can be improved upon by employing the following iterative refinement scheme (Fig. 5):

1. Estimate correlations using the basic procedure described above.
2. Identify the most strongly correlated pair of components that was not previously excluded. If the magnitude of this strongest correlation exceeds a given threshold, add this pair to the set of excluded pairs. Otherwise, terminate the estimation procedure.
3. Identify components that form only excluded pairs and completely exclude them from the analysis. Since the assumptions of our method are not met by such components, it is unable to infer their correlations. If all components but three are excluded, terminate the estimation procedure, as the sparsity assumption is violated for the whole system.
4. If any components were excluded, re-estimate the fractions of the remaining components. Note that the new fractions are relative to the new subset of components.
5. Calculate the component variations 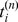, excluding all strongly correlated pairs. That is, if 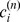 is the set of indices of components identified to be strongly correlated with component 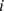 at the previous, 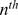, iteration, then
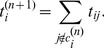
(15)
6. Use the newly computed component variations to compute the basis correlations, as in steps 4 and 5 of the basic inference procedure.
7. Repeat steps 2 through 6 for a given number of iterations, or until no new strongly correlated pairs are identified.

Note that the iterative procedure can result in correlations whose magnitude is greater than 1, indicating that too many pairs were excluded. Setting a higher exclusion threshold, or a lower iteration number will remedy this fallacy, though the resulting approximation is likely to be of poor accuracy.

Basis correlation can also be inferred using transformed variables (see Text S1). However, the iterative exclusion detailed above improves the quality of the approximation, making SparCC superior to these alternatives (Fig. S1)

To account for the sampling noise, the inference procedure is repeated multiple times, each time with fraction values drawn randomly from their posterior distribution, generating a distribution of each pairwise correlation. The median value of each pairwise correlation distribution is taken as its estimated value. In this work, a threshold of 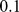 and a maximal number of 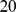 iterations were chosen, and the iterative procedure was repeated 100 times.

### Comparison of HMP networks inferred using Pearson and SparCC

For each body site, pairwise correlations between all OTUs were inferred using both Pearson and SparCC as described above. Interaction networks were subsequently build by connecting all OTU pairs that had a correlation magnitude greater than a given threshold. Results reported in the main text were obtained using a threshold value of 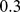. Comparison between corresponding Pearson and SparCC networks was done by treating the SparCC network as the true one, and computing the number of true-positives (TP), false-positives (FP), true-negatives (TN) and false-negatives (FN) detected in the Pearson network. The above quantities were calculated as following:
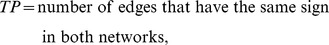


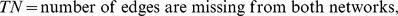


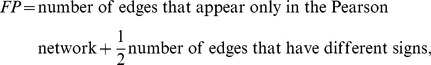


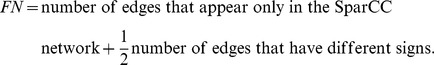



### Assessing statistical significance

The statistical significance of the inferred correlations can be assessed using a bootstrap procedure. First, a large number of simulated datasets, where all components are uncorrelated, are generated as described in Material and Methods. Next, correlations are inferred from each simulated dataset using SparCC with the same parameter setting as is used for the original data. Finally, for each component pair, pseudo p-values are assigned to be proportion of simulated data sets for which a correlation value at least as extreme as the one computed for the original data was obtained.

### Computer implementation

All analysis and procedures were implemented in Python, utilizing the Numpy [29] and Networkx [30] modules. Plotting was done using the Matplotlib [31] module.

## Supporting Information

Dataset S1 Correlation values for all HMP body sites inferred using both Pearson and SparCC from real and shuffled data.
(ZIP)

Figure S1 **Similar correlation networks are observed for real world vs. randomly shuffled bacterial abundance data.** Correlation networks based on 16S survey data collected as part of the Human Microbiome Project (HMP), inferred using Pearson correlations (left column), and SparCC (right column). Additionally, Pearson correlation networks were inferred from shuffled HMP data (middle column), where all OTUs are independent. This figures is extends Fig. 1 to include all 18 HMP body sites.
(PDF)

Figure S2 **Spearman correlations inference quality deteriorates with decreasing diversity.** Like Pearson correlations, Spearman correlations are also affected by the compositionally of the data and yield correlation networks that are only marginally more accurate than Pearson correlation networks (compare Fig. 2). Data simulation procedure and parameter values are identical to those used in Fig. 2.
(PDF)

Figure S3 **Root-mean-square error (RMSE) of both Spearman CLR inferred correlations.** The accuracy of Spearman correlations (A) is comparable to that of Pearson correlations. CLR correlations (B) are more accurate than both Pearson and Spearman correlation, but not as accurate as SparCC correlations (compare Fig. 3). Note that the Spearman correlations estimated from the fractions were compared to the true basis Spearman correlations, rather than Pearson correlations. Data simulation procedure and parameter values are identical to those used in Fig. 3.
(PDF)

Figure S4 **CLR correlations are strongly biased when a small number of components is analyzed.** RMSE of SparCC (A) and CLR (B) correlations for datasets composed of 5 components. Data is simulated as described in Materials and Methods section of main text.
(PDF)

Figure S5 **SparCC is more accurate than alternative correlations even when considering only the strongest detected correlations.** RMSE of SparCC, Pearson and Spearman correlations whose inferred magnitude exceeds a given threshold. Data is simulated as described in Materials and Methods section of main text. Note that the Spearman correlations estimated from the fractions were compared to the true basis Spearman correlations, rather than Pearson correlations.
(PDF)

Figure S6 **HMP correlation networks inferred using SparCC.** Networks inferred using SparCC from the same data as in Fig. 6. This figures is extends Fig. 4 to include all 18 HMP body sites.
(PDF)

Table S1 Accuracy of HMP Pearson networks compared to SparCC networks.
(DOC)

Table S2 Correlation between OTUs decreases with phylogenetic distance.
(DOC)

Text S1 Correlation inference using transformed variables.
(PDF)
