# Supplementary material for: Inferring Correlation Networks from Genomic Survey Data
Source: PLoS Comput Biol. 2012 Sep 20;8(9):e1002687. doi: 10.1371/journal.pcbi.1002687 (PMC3447976; doi:10.1371/journal.pcbi.1002687)
Supplement: Figure S1 — Similar correlation networks are observed for real world vs. randomly shuffled bacterial abundance data. Correlation networks based on 16S survey data collected as part of the Human Microbiome Project (HMP), inferred using Pearson correlations (left column), and SparCC (right column). Additionally, Pearson correlation networks were inferred from shuffled HMP data (middle column), where all OTUs are independent. This figures is extends Fig. 1 to include all 18 HMP body sites. (PDF) [file pcbi.1002687.s002.pdf]

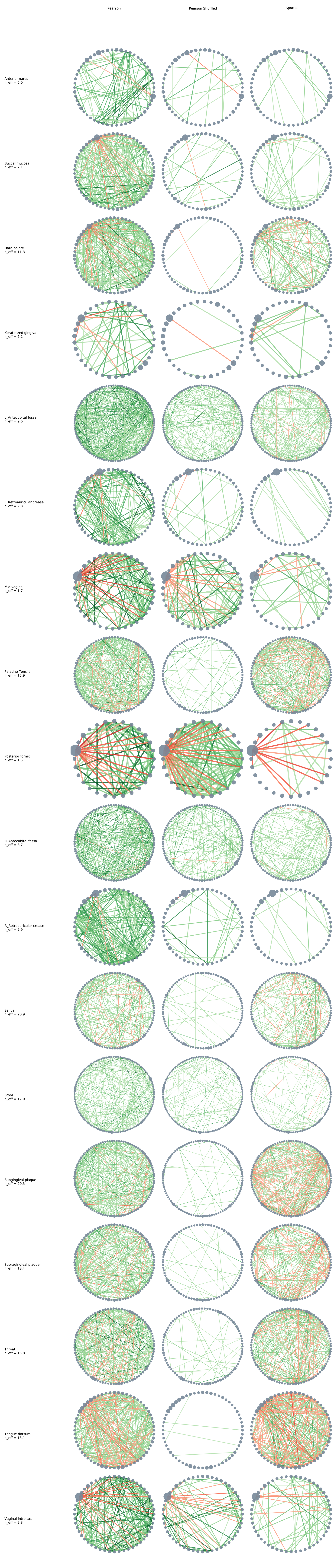

Pearson
Pearson Shuffled
SparCC
Anterior nares
n_eff = 5.0
Buccal mucosa
n_eff = 7.1
Hard palate
n_eff = 11.3
Keratinized gingiva
n_eff = 5.2
L_Antecubital fossa
n_eff = 9.6
L_Retroauricular crease
n_eff = 2.8
Mid vagina
n_eff = 1.7
Palatine Tonsils
n_eff = 15.9
Posterior fornix
n_eff = 1.5
R_Antecubital fossa
n_eff = 8.7
R_Retroauricular crease
n_eff = 2.9
Saliva
n_eff = 20.9
Stool
n_eff = 12.0
Subgingival plaque
n_eff = 20.5
Supragingival plaque
n_eff = 18.4
Throat
n_eff = 15.8
Tongue dorsum
n_eff = 13.1
Vaginal introitus
n_eff = 2.3
