# Supplementary material for: Inferring Correlation Networks from Genomic Survey Data
Source: PLoS Comput Biol. 2012 Sep 20;8(9):e1002687. doi: 10.1371/journal.pcbi.1002687 (PMC3447976; doi:10.1371/journal.pcbi.1002687)
Supplement: Figure S2 — Spearman correlations inference quality deteriorates with decreasing diversity. Like Pearson correlations, Spearman correlations are also affected by the compositionally of the data and yield correlation networks that are only marginally more accurate than Pearson correlation networks (compare Fig. 2). Data simulation procedure and parameter values are identical to those used in Fig. 2. (PDF) [file pcbi.1002687.s003.pdf]

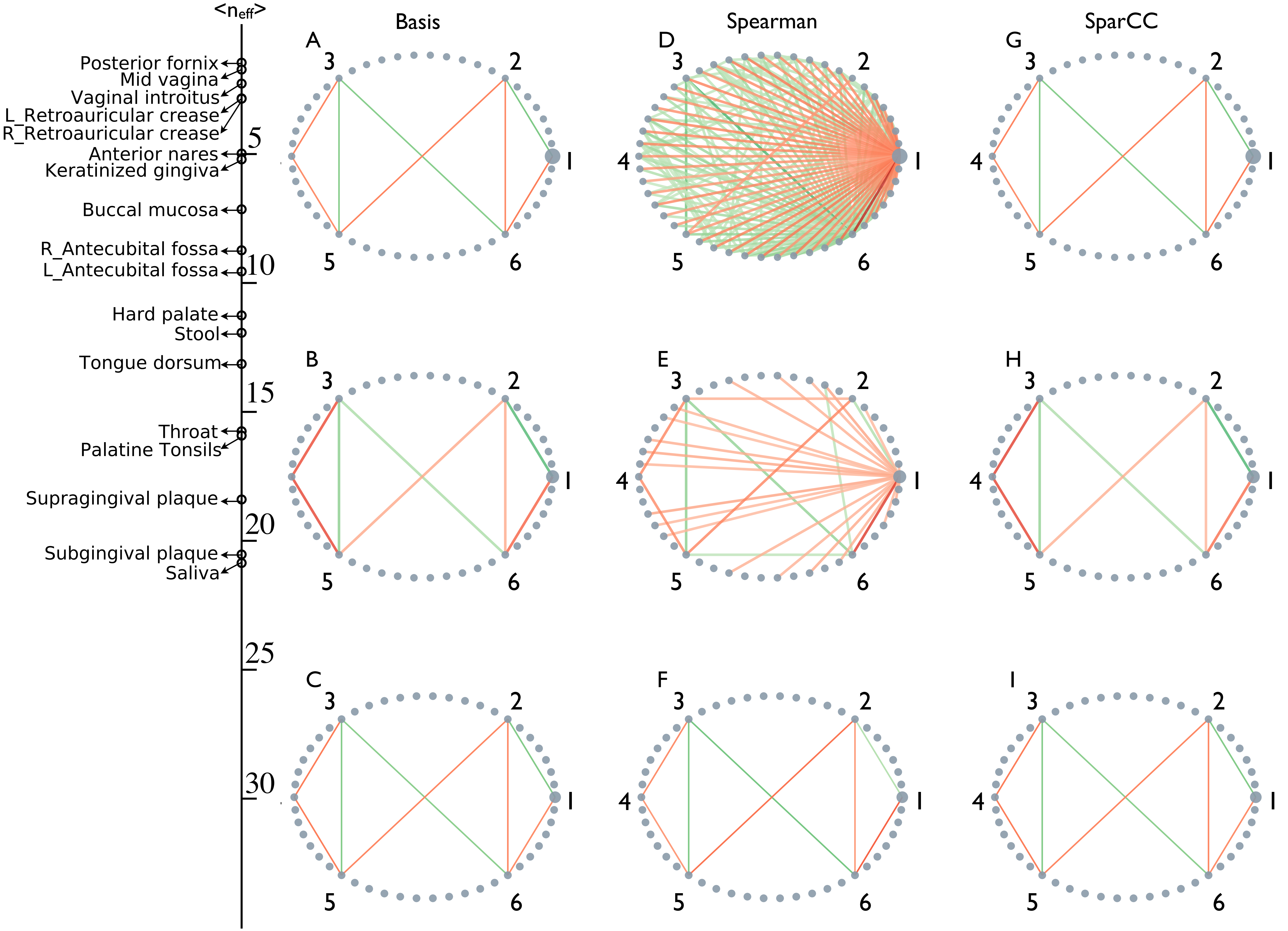

<n_eff>
Basis
Spearman
SparCC
A
B
C
D
E
F
G
H
I
Posterior fornix
Mid vagina
Vaginal introitus
L_Retroauricular crease
R_Retroauricular crease
Anterior nares
Keratinized gingiva
Buccal mucosa
R_Antecubital fossa
L_Antecubital fossa
Hard palate
Stool
Tongue dorsum
Throat
Palatine Tonsils
Supragingival plaque
Subgingival plaque
Saliva
5
10
15
20
25
30
1
2
3
4
5
6
