# Supplementary figures and images for: Inferring Correlation Networks from Genomic Survey Data
Source: PLoS Comput Biol. 2012 Sep 20;8(9):e1002687. doi: 10.1371/journal.pcbi.1002687 (PMC3447976; doi:10.1371/journal.pcbi.1002687)

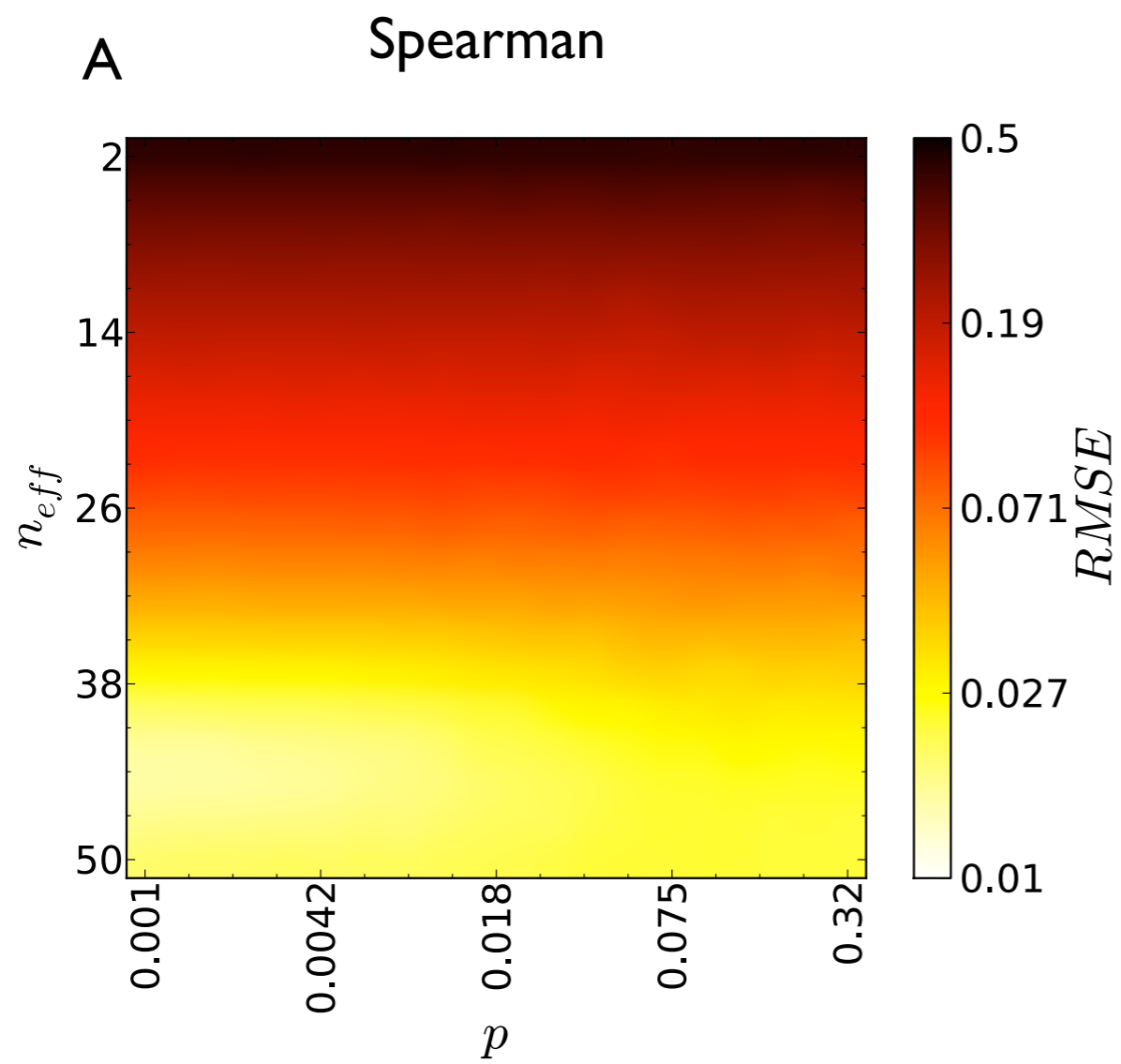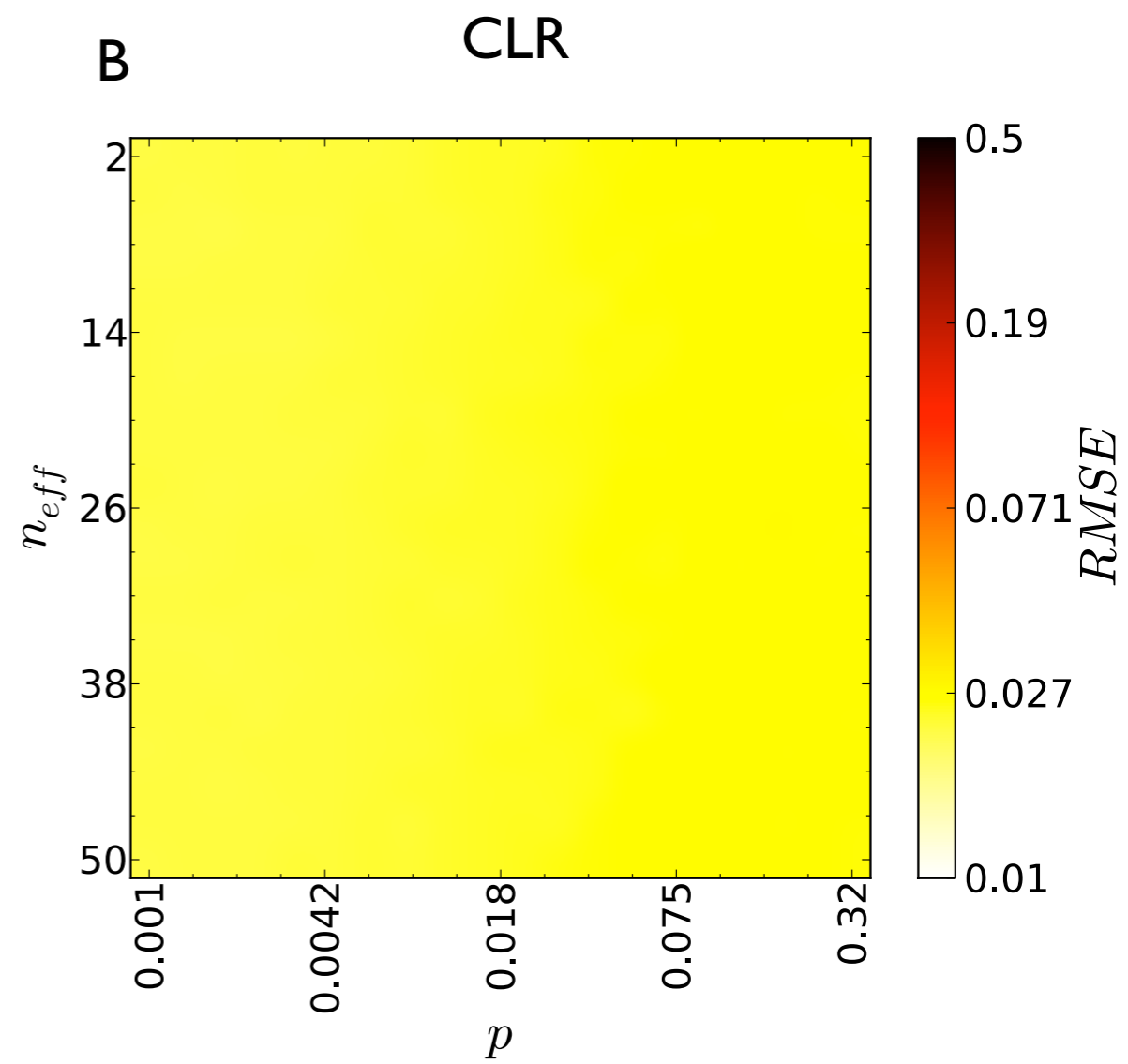

Supplement: Figure S3 — Root-mean-square error (RMSE) of both Spearman CLR inferred correlations. The accuracy of Spearman correlations (A) is comparable to that of Pearson correlations. CLR correlations (B) are more accurate than both Pearson and Spearman correlation, but not as accurate as SparCC correlations (compare Fig. 3). Note that the Spearman correlations estimated from the fractions were compared to the true basis Spearman correlations, rather than Pearson correlations. Data simulation procedure and parameter values are identical to those used in Fig. 3. (PDF) [file pcbi.1002687.s004.pdf]

**A****SparCC**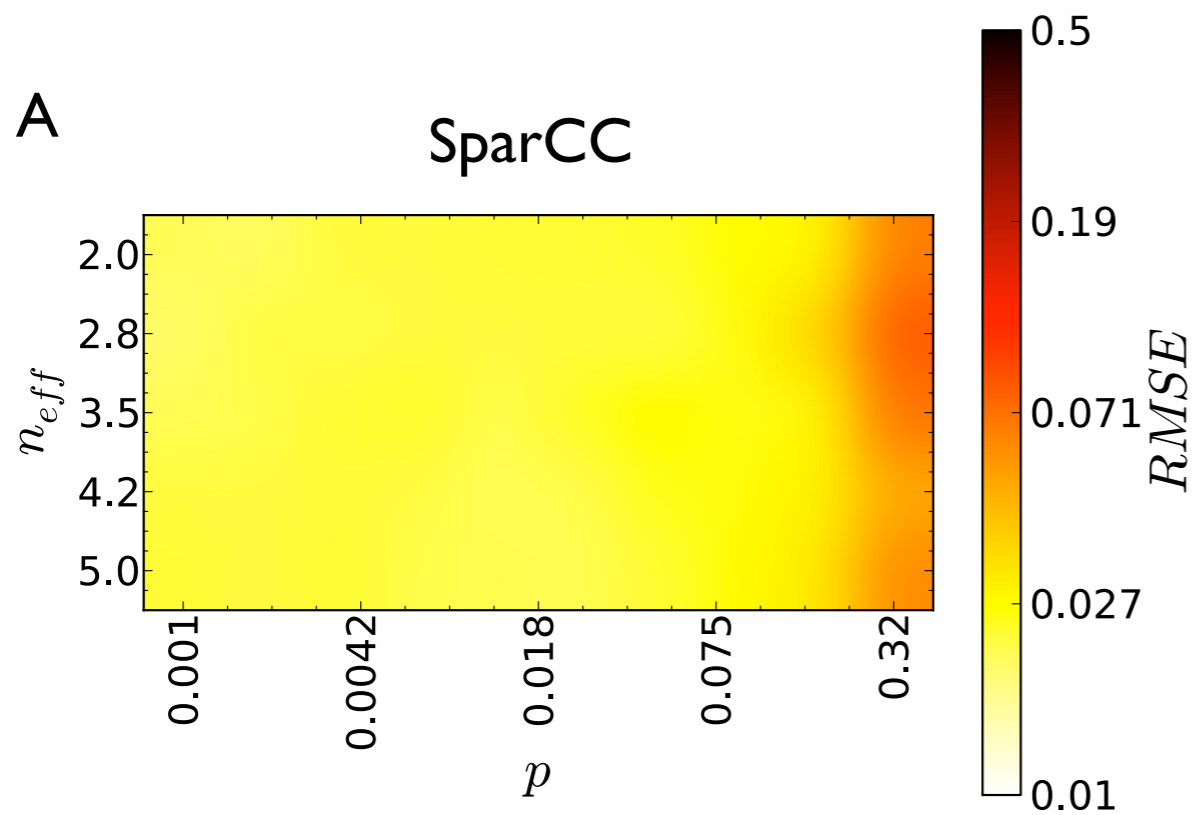**B****CLR**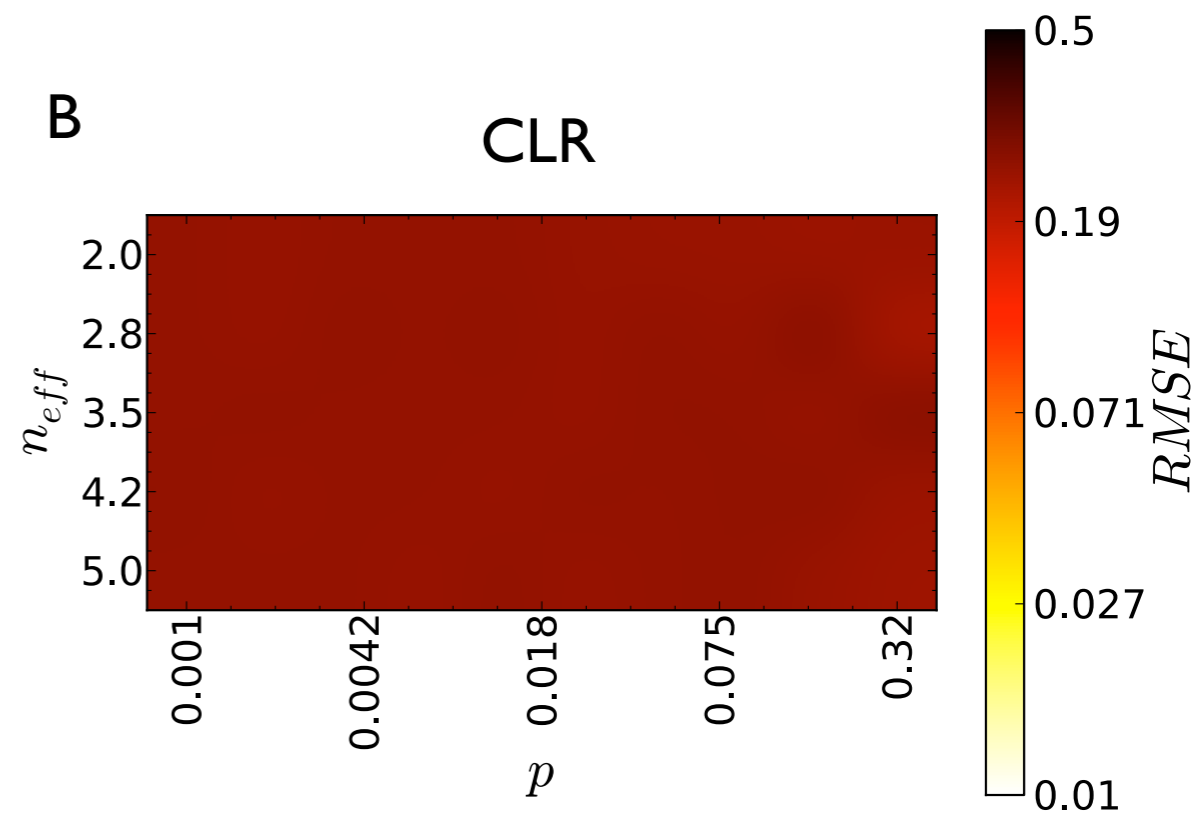

Supplement: Figure S4 — CLR correlations are strongly biased when a small number of components is analyzed. RMSE of SparCC (A) and CLR (B) correlations for datasets composed of 5 components. Data is simulated as described in Materials and Methods section of main text. (PDF) [file pcbi.1002687.s005.pdf]

$n_{\text{eff}} = 4$

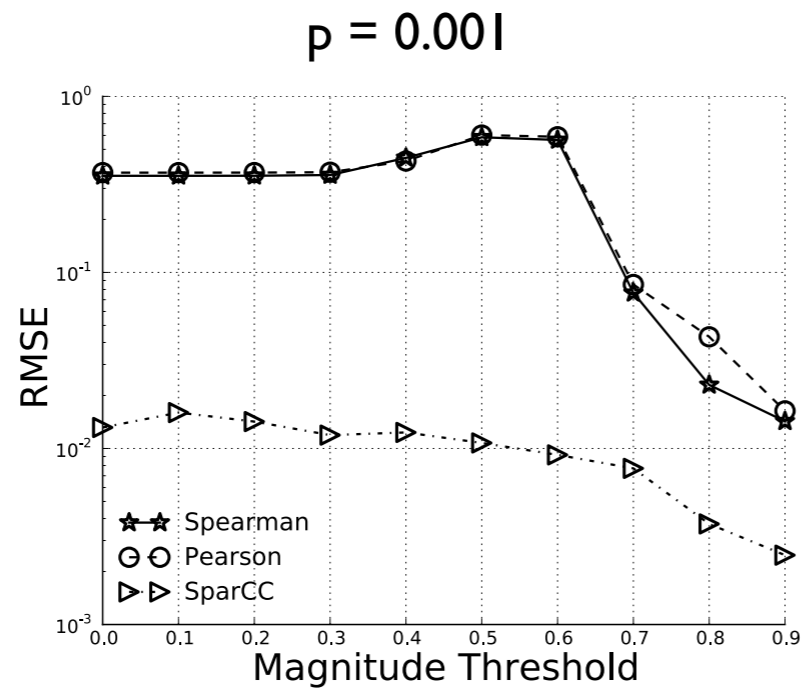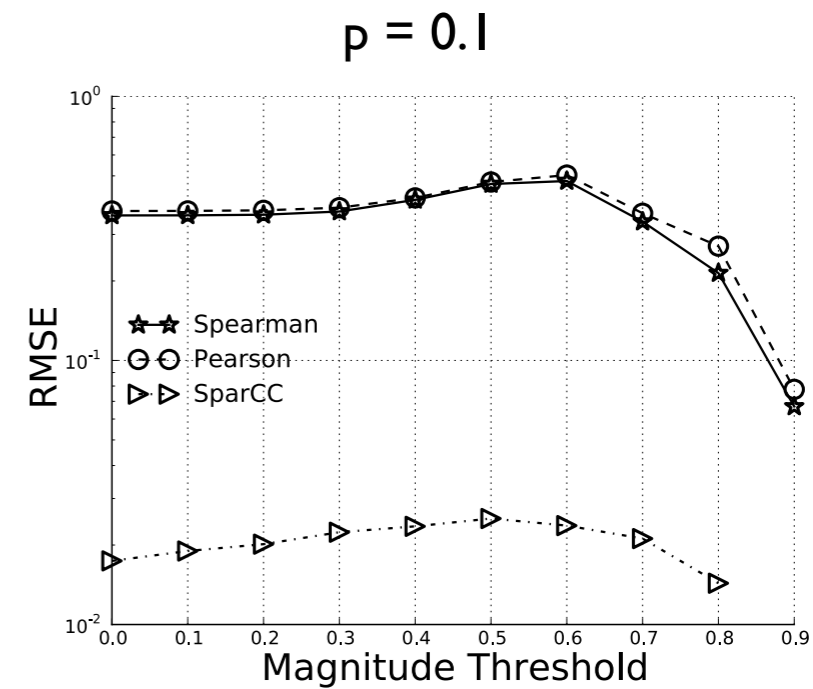

$n_{\text{eff}} = 14$

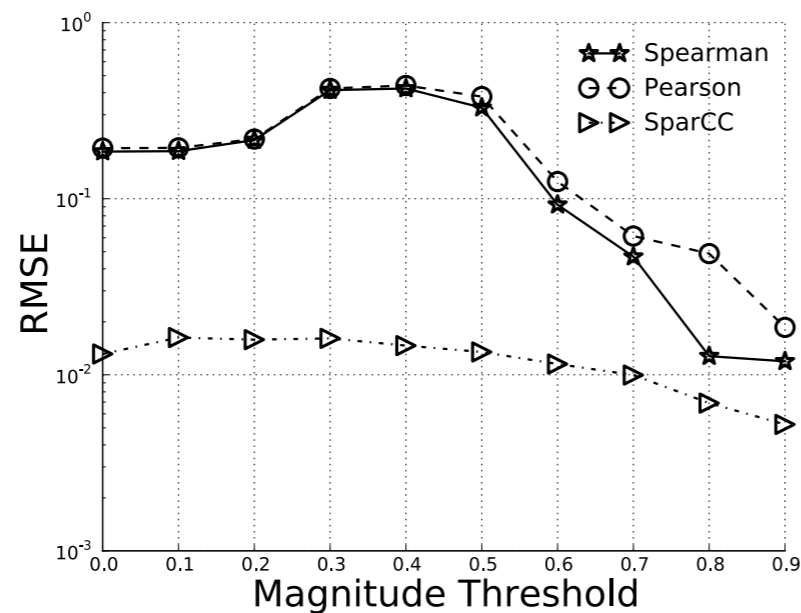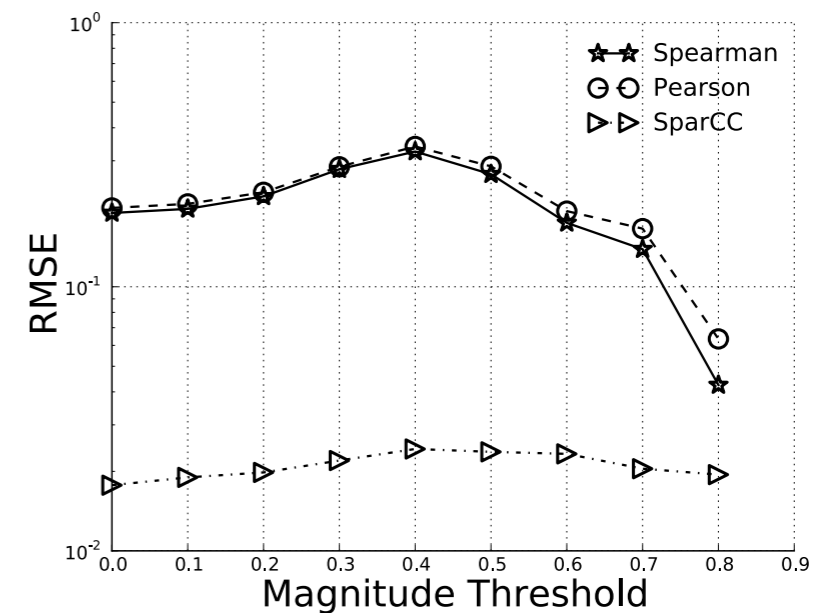

$n_{\text{eff}} = 24$

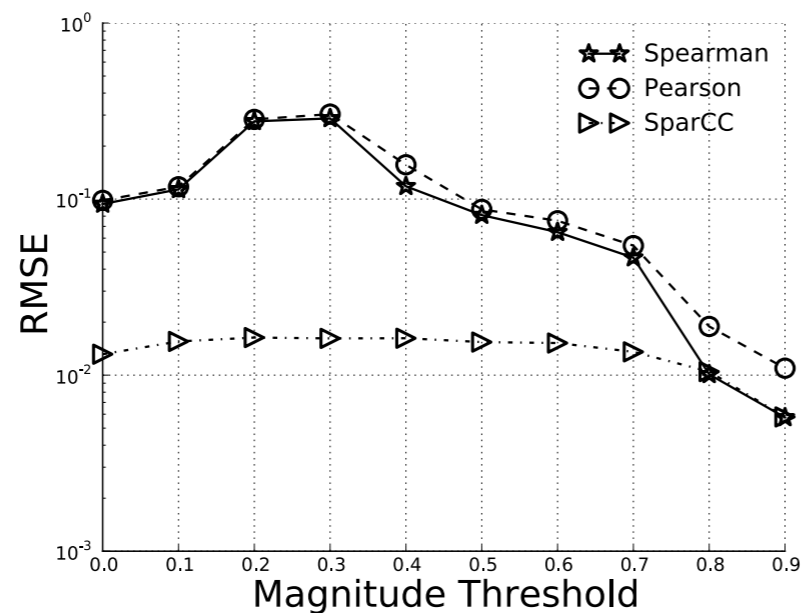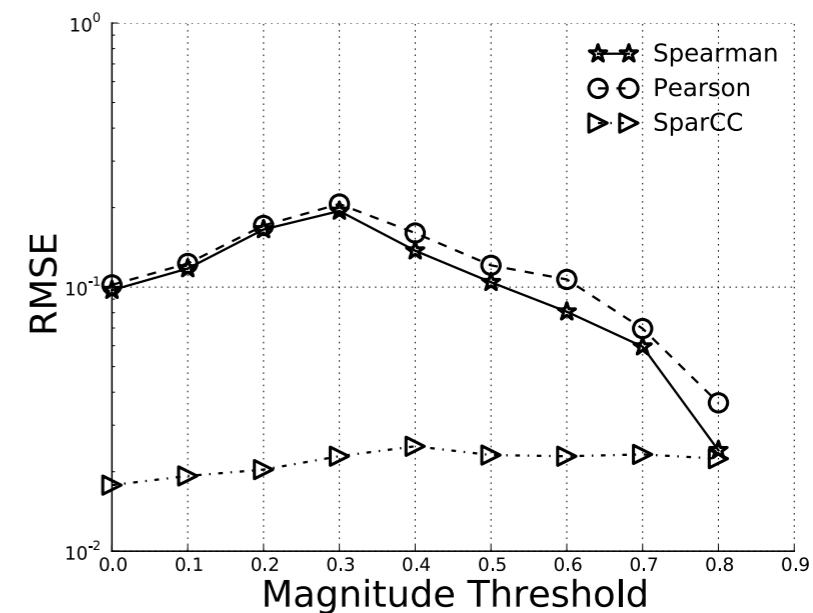

Supplement: Figure S5 — SparCC is more accurate than alternative correlations even when considering only the strongest detected correlations. RMSE of SparCC, Pearson and Spearman correlations whose inferred magnitude exceeds a given threshold. Data is simulated as described in Materials and Methods section of main text. Note that the Spearman correlations estimated from the fractions were compared to the true basis Spearman correlations, rather than Pearson correlations. (PDF) [file pcbi.1002687.s006.pdf]
