# Supplementary material for: Inferring Correlation Networks from Genomic Survey Data
Source: PLoS Comput Biol. 2012 Sep 20;8(9):e1002687. doi: 10.1371/journal.pcbi.1002687 (PMC3447976; doi:10.1371/journal.pcbi.1002687)
Supplement: Figure S6 — HMP correlation networks inferred using SparCC. Networks inferred using SparCC from the same data as in Fig. 6. This figures is extends Fig. 4 to include all 18 HMP body sites. (PDF) [file pcbi.1002687.s007.pdf]

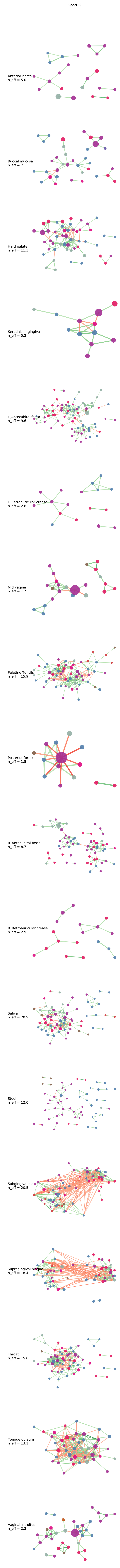
SparCC
Anterior nares
n_eff = 5.0
Buccal mucosa
n_eff = 7.1
Hard palate
n_eff = 11.3
Keratinized gingiva
n_eff = 5.2
L_Antecubital fossa
n_eff = 9.6
L_Retroauricular crease
n_eff = 2.8
Mid vagina
n_eff = 1.7
Palatine Tonsils
n_eff = 15.9
Posterior fornix
n_eff = 1.5
R_Antecubital fossa
n_eff = 8.7
R_Retroauricular crease
n_eff = 2.9
Saliva
n_eff = 20.9
Stool
n_eff = 12.0
Subgingival plaque
n_eff = 20.5
Supragingival plaque
n_eff = 18.4
Throat
n_eff = 15.8
Tongue dorsum
n_eff = 13.1
Vaginal introitus
n_eff = 2.3
