# Supplementary material for: Inferring Correlation Networks from Genomic Survey Data
Source: PLoS Comput Biol. 2012 Sep 20;8(9):e1002687. doi: 10.1371/journal.pcbi.1002687 (PMC3447976; doi:10.1371/journal.pcbi.1002687)
Supplement: Table S1 — Accuracy of HMP Pearson networks compared to SparCC networks. (DOC) [file pcbi.1002687.s008.doc]

Table S1: Accuracy of HMP Pearson networks compared to SparCC networks.

| **site** | **TP** | **TN** | **FP** | **FN** | **Precision (PPV)** | **Recall (TPR)** |
| --- | --- | --- | --- | --- | --- | --- |
| Anterior nares | 11 | 1338 | 76 | 6 | 0.13 | 0.65 |
| Buccal mucosa | 26 | 1483 | 188 | 14 | 0.12 | 0.65 |
| Hard palate | 85 | 1723 | 172 | 36 | 0.33 | 0.7 |
| Keratinized gingiva | 8 | 612 | 35 | 11 | 0.19 | 0.42 |
| L_Antecubital fossa | 142 | 5493 | 290 | 70 | 0.33 | 0.67 |
| L_Retroauricular crease | 16 | 1328 | 140 | 1 | 0.1 | 0.94 |
| Mid vagina | 30 | 623 | 84 | 4 | 0.26 | 0.88 |
| Palatine Tonsils | 111 | 3465 | 197 | 143 | 0.36 | 0.44 |
| Posterior fornix | 21 | 304 | 53 | 0 | 0.28 | 1 |
| R_Antecubital fossa | 83 | 4514 | 207 | 47 | 0.29 | 0.64 |
| R_Retroauricular crease | 14 | 1126 | 186 | 0 | 0.07 | 1 |
| Saliva | 70 | 4379 | 119 | 88 | 0.37 | 0.44 |
| Stool | 29 | 9730 | 211 | 41 | 0.12 | 0.41 |
| Subgingival plaque | 119 | 5237 | 193 | 229 | 0.38 | 0.34 |
| Supragingival plaque | 75 | 3435 | 193 | 125 | 0.28 | 0.38 |
| Throat | 120 | 3690 | 155 | 130 | 0.44 | 0.48 |
| Tongue dorsum | 130 | 1390 | 129 | 121 | 0.5 | 0.52 |
| Vaginal introitus | 46 | 1145 | 123 | 12 | 0.27 | 0.79 |
