# Supplementary material for: Inferring Correlation Networks from Genomic Survey Data
Source: PLoS Comput Biol. 2012 Sep 20;8(9):e1002687. doi: 10.1371/journal.pcbi.1002687 (PMC3447976; doi:10.1371/journal.pcbi.1002687)
Supplement: Table S2 — Correlation between OTUs decreases with phylogenetic distance. (DOC) [file pcbi.1002687.s009.doc]

Table S2: Correlation between OTUs decreases with phylogenetic distance.

| **site** | **Spearman r** | **p-value** |
| --- | --- | --- |
| Anteriornares | -0.06 | 3.70E-02 |
| Buccalmucosa | -0.15 | 1.70E-08 |
| Hardpalate | -0.06 | 5.70E-03 |
| Keratinizedgingiva | -0.09 | 2.10E-02 |
| LAntecubitalfossa | -0.09 | 2.60E-10 |
| LRetroauricularcrease | -0.09 | 2.00E-03 |
| Midvagina | -0.06 | 1.10E-01 |
| PalatineTonsils | -0.05 | 7.70E-03 |
| Posteriorfornix | -0.19 | 1.30E-03 |
| RAntecubitalfossa | -0.09 | 4.60E-09 |
| RRetroauricularcrease | -0.08 | 1.00E-02 |
| Saliva | -0.1 | 1.70E-10 |
| Stool | -0.17 | 3.20E-49 |
| Subgingivalplaque | -0.09 | 1.00E-08 |
| Supragingivalplaque | -0.09 | 7.30E-07 |
| Throat | -0.03 | 6.70E-02 |
| Tonguedorsum | -0.11 | 1.30E-05 |
| Vaginalintroitus | -0.1 | 3.50E-04 |
