# Supplementary material for: Inferring Correlation Networks from Genomic Survey Data
Source: PLoS Comput Biol. 2012 Sep 20;8(9):e1002687. doi: 10.1371/journal.pcbi.1002687 (PMC3447976; doi:10.1371/journal.pcbi.1002687)
Supplement: Text S1 — Correlation inference using transformed variables. (PDF) [file pcbi.1002687.s010.pdf]

Inferring Correlation Networks from Genomic Survey Data: Supplementary Text

Jonathan Friedman[1], Eric J. Alm[1,2,3,*]
1 Computational & Systems Biology Initiative, Massachusetts Institute of Technology, Cambridge, Massachusetts 02139, USA
2 Departments of Biological Engineering & Civil and Environmental Engineering, Massachusetts Institute of Technology, Cambridge, Massachusetts 02139, USA
3 The Broad Institute, Cambridge, Massachusetts 02140, USA
∗ E-mail: ejalm@mit.edu

Correlation inference using transformed variables

In SparCC, we utilize the variation matrix to capture the variability of component compositions. Aitchison showed that the same variability information can be equivalently represented by the covariance matrices of log-ratio transformed variables [1], thus one may expect these covariance matrices to also serve as departure points for basis correlations calculation. In the rest of this section we describe how information regarding the basis correlations can be extracted from the covariance matrices of fractions transformed using Aitchison's additive log ratio (alr) and central log ratio (clr) transformations.

The alr covariance matrix allows detection of situations when all components are independent through Aitchison's test of complete subcompositional independence. In the alr transformation, a D-dimensional set of fraction $(x_1, x_2, ..., x_D)$ is transformed to a $(D-1)$-dimensional set of variables $(y_1, y_2, ..., y_{D-1})$ by setting:

$$y_i = \log \frac{x_i}{x_D} \quad i = \{1, 2, \ldots, D-1\}. \tag{1}$$

Aitchinson showed that when all components are independent the alr covariance matrix takes the special form:

$$\mathrm{Cov}(\underline{y}) = \begin{pmatrix} \omega_1^2 + \omega_D^2 & & & \Large\omega_D^2 \\ & \omega_2^2 + \omega_D^2 & & \\ & & \ddots & \\ \Large\omega_D^2 & & & \omega_{D-1}^2 + \omega_D^2 \end{pmatrix},$$

and constructed a test of complete subcompositional independence, in which the null hypothesis is the the alr covariance matrix takes the above form, and the alternative is that the alr covariance matrix takes a general positive semi-definite form [2], [3]. Although this test is statistically sound, and results in exact p-values, rejection of the null hypothesis only indicates the existence of at least one pair of correlated components, but it does not indicate which components are correlated, or what is the magnitude of the correlation.

When there are many, sparsely correlated component, basis correlations are approximately equal to the correlations between clr transformed variables. The clr transformation is defined as:

$$z_i = \log \frac{x_i}{g(\underline{x})} = \log \frac{w_i}{g(\underline{w})} \quad i = \{1, 2, \ldots, D\}, \tag{2}$$

where $g(\underline{x})$ is the geometric mean of the component values in a sample, i.e.

$$g(\underline{x}) = \sqrt[D]{\prod_i^D x_i}. \tag{3}$$

The relation between covariance of clr transformed variables and the basis covariance is given by

$$\begin{aligned}
\mathrm{Cov}(z_i, z_j) &= \mathrm{Cov}\left[\log\frac{w_i}{g(\underline{w})}, \log\frac{w_j}{g(\underline{w})}\right] = \mathrm{Cov}\left[\log w_i - \log g(\underline{w}), \log w_j - \log g(\underline{w})\right] \\
&= \mathrm{Cov}\left[\log w_i, \log w_j\right] - \frac{1}{D}\sum_{n=1}^{D}\mathrm{Cov}\left[\log w_i, \log w_n\right] \\
&\quad - \frac{1}{D}\sum_{m=1}^{D}\mathrm{Cov}\left[\log w_j, \log w_m\right] + \frac{1}{D^2}\sum_{n=1}^{D}\sum_{m=1}^{D}\mathrm{Cov}\left[\log w_n, \log w_m\right] \\
&\equiv \omega_i\omega_j\rho_{ij} - \omega_i\langle\omega_n\rho_{in}\rangle_n - \omega_j\langle\omega_m\rho_{jm}\rangle_m + \langle\omega_n\omega_m\rho_{nm}\rangle_{nm}.
\end{aligned} \tag{4}$$

Consider again the simpler case where all basis variables have the same variance ω. Eq. 4 simplifies to

$$\mathrm{Cov}(z_i, z_j) = \omega^2\left[\rho_{ij} + \frac{D-1}{D}\Big(\langle\rho\rangle - \langle\rho_i\rangle - \langle\rho_j\rangle\Big) - \frac{1}{D}\right], \tag{5}$$

where the averaging is over correlation between different components (the term $1/D$ captures the self correlations). It is now clear that when the network is sparse, and has many components, the correction terms to the basis correlation ρ_{ij} will be small. It is worth noting that though the basis correlation itself may often be small, and the correction not negligible, no large spurious correlations will be induced. For example, if all components are independent, the correlation between clr transformed variables will be

$$\mathrm{Corr}(z_i, z_j) = \frac{-\omega^2/D}{\omega^2(D-1)/D} = -\frac{1}{D-1}. \tag{6}$$

Eq. (6) demonstrates that CLR based correlations are negatively biased, and the size of the bias is inversely proportional to the number of components. Thus, correlations inferred from a small number of components will be severely skewed (Fig. S4). Another advantage of using the variation matrix over the clr covariance matrix is that it enables the exclusion of strongly correlated component pairs, employed in the iterative SparCC procedure, thus improving the approximation quality (see Fig. S3A for clr based inference quality.). The advantage of the clr based inference is it's simplicity and scalability (unlike SparCC, it does not require inverting a potentially large matrix.), which make it particularly useful for analyzing large, diverse datasets, for which it's accuracy is similar to that of SparCC.

References

1. Aitchison J (2003) The statistical analysis of compositional data. Caldwell, New Jersey, USA: Blackburn Press, 416 pp.

2. Aitchison J (1981) A new approach to null correlations of proportions. Math Geol 13: 175–189.

3. Woronow A, Butler J (1986) Complete subcompositional independence testing of closed arrays. Computers & Geosciences 12: 267–279.
